# Linguistic validation of the Sexual Inhibition and Sexual Excitation Scales (SIS/SES) translated into five South Asian languages: Oxford Sexual Dysfunction Study (OSDS)

**DOI:** 10.1186/1756-0500-6-550

**Published:** 2013-12-20

**Authors:** Lasantha S Malavige, Pabasi N Wijesekara, Shanthilal D Jayaratne, Samudra T Kathriarachchi, Priyanga Ranasinghe, Sivagurunathan Sivayogan, Jonathan C Levy, John Bancroft

**Affiliations:** 1 Nuffield Department of Clinical Medicine, University of Oxford, Oxford, UK; 2 Department of Medicine, Faculty of Medical Sciences, University of Sri Jayawardenapura, Colombo, Sri Lanka; 3 Department of Psychiatry, Faculty of Medical Sciences, University of Sri Jayawardenapura, Colombo, Sri Lanka; 4 Department of Pharmacology, Faculty of Medicine, University of Colombo, Colombo, Sri Lanka; 5 Department of Community Medicine, Faculty of Medicine, University of Sri Jayawardenapura, Colombo, Sri Lanka; 6 Oxford Centre for Diabetes, Endocrinology and Metabolism, Oxford Radcliffe NHS Trust, Oxford, UK; 7 Indiana University, The Kinsey Institute for Research in Sex, Gender and Reproduction, Indiana, USA

**Keywords:** Linguistic validation, Sexual excitation, Sexual inhibition, Cross cultural comparison, Cultural differences, Desire

## Abstract

**Background:**

The purpose of the linguistic validation of the Sexual Inhibition and Sexual Excitation Scales (SIS/SES) was to produce translated versions in five South Asian languages (Hindi, Urdu, Panjabi, Tamil and Sinhalese) that was “conceptually equivalent” to the original U.S. English version, for use in the Oxford Sexual Dysfunction Study (OSDS).

**Methods:**

Initially an expert committee was appointed to carry out the task of linguistic validation. This committee included the principal investigator, project coordinator and the associate project manager of the OSDS and a language consultant for each of the South Asian languages. The process of translation and validation was conducted in the following order; a) production of two independent forward translations, b) comparison and reconciliation of the translations, c) backward translation of the first reconciled version, d) comparison of the original version of SIS/SES and the backward version leading to the production of the second reconciled version and e) pilot testing and finalization.

**Results:**

Several linguistic and conceptual issues arose during the process of translating the instrument. Problems were also encountered with cultural differences in acceptability of certain concepts, and with semantic difficulties in finding an appropriate translation. In addition, the researchers had to find culturally acceptable equivalents for some terms and idiomatic phrases. The problems encountered in pilot testing, during cognitive debriefing and clinicians’ review, were categorized as cultural or conceptual/semantic. Cultural issues describe the acceptability of using certain terms and phrases in a particular socio-cultural milieu. The conceptual and semantic difficulties reflect the inability to deliver the idea/meaning of a source statement in the target language. The current paper describes a selection of these issues.

**Conclusions:**

We applied a rigorous translation method to ensure conceptual equivalence and acceptability of SIS/SES in the five different South Asian languages prior to its utilization in the OSDS. However, to complete the cultural adaptation process, future psychometric validation of the translated versions is required among the different language speakers.

## Background

There are striking differences in sexual attitudes, values and behaviours among different ethnic groups, even among those established in the United Kingdom (UK) [1-3]. For example, in a well conducted study among Black Caribbean, Caucasian, Indian and Pakistani men living in the UK, the mean number of life time sexual partners were 23.9, 12.5, 5.2 and 7.4 respectively, while the percentages ever diagnosed with sexually transmitted infections were 19.7, 10.9, 3.4 and 3.2 respectively [1]. Ethnic differences in sexual dysfunction have also been reported. A significantly higher proportion of men from Muslim countries living in the West have sought help for premature ejaculation compared to men from other ethnic and religious backgrounds [3-5]. In an east London clinic, 84% of the men seeking help for premature ejaculation were of Bangladeshi origin, despite the percentage of men from this ethnic background in the catchment area being only 33.4% [3]. However, the reasons for such differences are not clearly understood. Furthermore, attitudes to sex and the perceived role of sexual activity are very strongly influenced by cultural values and culturally determined gender roles influence relationships between different-sex partners, while cultural values also affect attitudes towards sexual variation [6].

The Oxford Sexual Dysfunction Study (OSDS) is an ongoing project in which participants include men of European and South Asian origin living in the United Kingdom (UK) [7]. Individuals whose parents are from India, Pakistan, Bangladesh, Sri Lanka, Bhutan or Maldives are considered South Asian in this study. One of the objectives of this project was to assess the impact of ethnicity and culture on sexual arousal (erectile function) and ejaculatory function. For this purpose it was decided to use the SIS/SES scales to measure the propensity for sexual excitation and inhibition, an instrument derived from the Dual Control Model of sexual response [8].

The SIS/SES scales were developed for measuring individual propensities for sexual excitation and sexual inhibition, and they have been shown to have good reliability and validity. The questionnaire is comprised of 45 items and each item is rated on a four point scale; 1 = ‘strongly agree’, 2 = ‘agree’, 3 = ‘disagree’ and 4 = ‘strongly disagree’. Sexual excitation consists of a single scale and sexual inhibition consists of two scales, Inhibition Due to Threat of Performance Failure (SIS-1) and Inhibition Due to Threat of Performance Consequences (SIS-2). Together these three scales have shown a close to normal distribution of scores in non-clinical studies, with the middle range of each scale reflecting a normative or adaptive response, and the high and low ends of each scale being potentially problematic [9]. However, the extent to which propensities for sexual excitation and sexual inhibition vary across cultures has not yet been reported, although the impact of culture on sexual attitudes and behaviours is well accepted. It is hoped that the use of the SIS/SES scales in the OSDS will give us the opportunity to assess the effect of culture, attitudes and values on an individuals’ sexual arousability. However, for this purpose it is necessary to translate the SIS/SES scales into the relevant South Asian languages.

Five languages commonly used by South Asians living in the UK were selected as the target languages for translation of the measures used; namely, Hindi, Urdu, Panjabi, Tamil and Sinhalese. Since the OSDS involves comparison of groups with marked socio-cultural differences, it is important that the language versions of the various measures used, including the SIS/SES, are conceptually equivalent to one another and to the original, as well as being culturally relevant and acceptable to each target socio-cultural group. The scientific process of translation for this purpose is known as linguistic validation and cultural adaptation. In this paper we report the methodology used, the problems encountered and the solutions obtained in the linguistic validation process of the SIS/SES.

## Method

**Figure 1:**
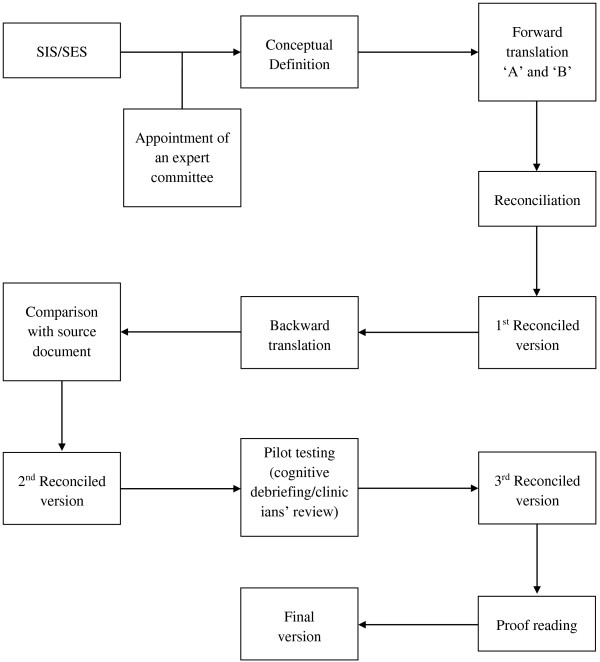
Algorithm of the linguistic validation processes.

The linguistic validation process was carried out using a previously established and scientifically accepted method [10]. Ethical approval for the study was obtained from Oxfordshire Research Ethics Committee C and study was conducted in accordance with the Declaration of Helsinki. Informed written consent was obtained from all participants involved in the pilot testing of the questionnaire.The language used in the original study instrument (United States English), is referred to as the “source language” and the language to which the study instrument is translated to is referred to as the “target language”. A schematic presentation of the entire validation process used in this study is illustrated in Figure 1. The six step process used in the linguistic validation was as follows;

1. Appointment of an expert committee

Initially an expert committee was appointed to carry out the task of linguistic validation. The expert committee included the principal investigator, project coordinator, the associate project manager of the OSDS and a consultant for each of the five South Asian languages. The consultant was fluent in the source language and a native speaker of the target language.

2. Conceptual Definition

The original instrument was analysed in collaboration with the developer of the original SIS/SES and the basic concept in each question was clarified to ensure that it is reflected correctly in the target language versions.

3. Forward translation

For each of the South Asian languages two translations of the instrument were carried out independently by two professional translators. The translators were native speakers of the target language and fluent in the source language. Subsequently a meeting between the two forward translators took place in collaboration with a member of the expert committee to produce the first reconciled version of the forward translation.

4. Backward translation

The first reconciled version was translated back into the source language by an independent translator, who was a native speaker of the source language and was also fluent in the target language. These back translations were compared with the source documents by the expert committee to check the conceptual content and relevant amendments were made, after a detailed discussion with the back translators and the forward translators, resulting in the second reconciled version.

5. Pilot testing

This was carried out in two simultaneous and parallel steps;

a) Cognitive debriefing – For this purpose an attempt was made to include volunteers from different age groups and a combination of men living in the respective native countries as well as in UK, with a view of making the translation acceptable to a broader group of men. Structured interviews were performed on five individuals per language, using the second reconciled version of the study instrument, with the aim of assessing the meaning, clarity, absence of ambiguity, ease of comprehension, appropriateness (neither too colloquial nor too sophisticated) and the cultural relevance and acceptability of the translated version. If problems related to any of the above issues were identified they were recorded, together with suggestions for improvement.

b) Clinicians’ review - The second reconciled version for each of the five languages were reviewed by a clinician who is a native speaker of the target language and fluent in the source language, with regard to clinical acceptability and use of terminology. The clinician’s comments were recorded. The cognitive debriefing and clinician’s review comments were reviewed by the expert committee and the translators, in order to make the third version of the study instrument.

6. Proof Reading

The third version was proof-read by the expert committee and the translators, and final versions for each of the five target languages were obtained.

## Results

The mean age of the 25 volunteers recruited for cognitive debriefing (five per language) was 40.7 years and the age range was 22 to 68 years, 64% of the men were married and 92% had completed their secondary education. The problems encountered in pilot testing, during cognitive debriefing and clinicians’ review, were categorized as cultural or conceptual/semantic. Cultural issues describe the acceptability of using certain terms and phrases in a particular socio-cultural milieu. The conceptual and semantic difficulties reflect the inability to deliver the idea/meaning of a source statement in a target language.

### Cultural issues

Translating the terms “partner/sexual partner” used widely in the source document (item 17, 21, 34, 45), raised concerns of cultural acceptability by 8 out of 25 men who suggested “wife” instead of “sexual partner”. This issue was further compounded in Tamil and Sinhalese due to the non-availability of a common term to describe sexual partner of either sex, therefore, the translation of “male or female partner” was used. The expert committee decided that the translation of “sexual partner” in Hindi, Urdu and Panjabi versions and translation of “male or female sexual partner” in Tamil and Sinhalese versions should be used to maintain consistency with the conceptual definition of the source document.

The third item of the source questionnaire is “Sometimes I become sexually aroused just by lying in the sun”. As lying in the sun is not a common leisure time activity in most South Asian countries, this was considered culturally irrelevant by 6 of the 25 participants, five of whom were living in their native countries. However, this statement was retained to maintain consistency of the scale and due to the fact that the OSDS is to be conducted in UK.

Item 7 of the SIS/SES is “When I have a quiet candlelight dinner with someone I find sexually attractive, I become sexually aroused”. The exact translation of “candle light dinner” did not convey the meaning of a romantic experience to 7 of the 25 participants. “Dinner under dim light on a quiet and calm night” was considered a better phrase. In the Hindi version; the word “romantic” was mentioned within brackets as this is an English term commonly used by the Hindi speaking community.

### Conceptual/semantic issues

Specific terms with reasonable socio-cultural acceptability to describe sexual behaviours were lacking in most of the target language vocabularies. Many terms available were either too colloquial or too sophisticated making them difficult to be understood by a broader group of men. Therefore, during the validation process many terms used were revised to make them more acceptable and understandable. Some of the more interesting conceptual and semantic issues encountered are reported here.

In the source document “hot”, “horny” and “turned on” were used to describe sexual excitation. It was difficult to find acceptable synonyms in most of the South Asian languages. Most of the words available to describe sexual excitation were considered either slang or too sophisticated. In some instances simple translations of instructions in the source document sounded offensive to the reader. For example, Urdu translation of “Try not to skip any questions”; “koi sawal chornai ki koshish na karain” was considered too offensive by 3 of 5 Urdu speakers and was changed to “koi sawal bhi na chorain” which was more of a request. The translation of “be honest”, “raast baaz”, was replaced by “diyanat daar” for the same reason. These changes were approved by the expert committee. Item 2 of the SIS/SES is “If I feel I am being rushed, I am unlikely to get very aroused”. In the Sinhalese version, translation of “being rushed” was unclear for two of the five native speakers. Changing the original question to “if I feel that I am being rushed in the sexual act by my male/female partner” and translating it as “mage sahakaruva/sahakaariya visin lingika kriyavehi maa hadisi karavana bava danei nam….” was proposed, and was accepted by the expert committee.

Item 6 of the SIS/SES is “When a sexually attractive stranger accidentally touches me, I easily become aroused”. The Tamil translation of this item resulted in ambiguity of the word used for ‘accidentally’. “Thatseyalaha”, which specifically means “accidentally”, was preferred to “thavaruthalaha” which can be used to describe both “accidentally” or “purposely” depending on the circumstances. The original translation of item 9 in Urdu, “I need my penis to be touched to maintain an erection”, as “kai koi mairai zikhr ko choohai” meant that “the penis is touched by someone else” to three of the five native speakers. The source statement does not specify the person who does the action and hence “…zuroori hai kai mairai zikarko chooha jai” was adopted to give the exact meaning of the original SIS/SES. Item 15 is “If I discovered that someone I find sexually attractive is too young, I would have difficulty getting sexually aroused with him/her”. In Hindi, translating “too young” as “choota” also meant ‘small in size’ or ‘short’ to three of the five men. The translation of the phrase “small in age” as “umr me bahut choota” was therefore adopted in the Hindi version by the expert committee.

Item 29 of the SIS/SES is “If I am with a group of people watching an X-rated film, I quickly become sexually aroused”. The original Sinhalese translation was “…… narambana pirisak athara sitina vitadee…..”. Two out of five men were unclear whether this meant watching the film as a team process or simply being with a group watching an X-rated film, but the individual not necessarily watching the film. Therefore, “maa pirisak samaga …….narambana vitadee” was adopted as a more appropriate translation by the expert committee as this makes it clear that it refers to someone actually watching the film together with a group of others. Item 37 is “When I start fantasizing about sex, I quickly become sexually aroused”. In the original, the term “sex” means “any sexual activity”. In Urdu the direct, translation of “sex” as “jins” meant “gender” to some men. It was therefore replaced with “jinsi amal” which explains that the person is thinking about “any sexual activity”. In the Tamil version, the phrase “udaluravu kolluthalai” was interpreted as “the act of coitus” by two of the five Tamil participants and was replaced with “paaliyal vidayankalai” which means any kind of sexual activity. A similar issue was encountered with the Sinhala language translation and was corrected. Both the Sinhalese and Tamil languages lacked simple equivalents for the words “fantasies” and “flirt” in items 41 and 44 respectively. The terms available for use were considered too sophisticated and difficult to understand by the participants. To overcome this the English terms “fantasies” and “flirting” were included within brackets as these words are commonly used among Sinhalese and Tamil speakers.

## Discussion

Different cultures, together with other factors, are characterised by people speaking different languages. Cross-cultural studies therefore need valid language versions of the questionnaires in the target language. This process is particularly important in the study of sexuality, which is associated with many culture- and faith- related taboos and sensitivities. These difficulties were compounded in the present study as most of the terms describing sexuality-related issues in South Asian languages were either colloquial or slang. The proper words used by the translators were found to be too difficult to be understood by an average man.

Ethnic groups are generally characterised by a sense of belonging or group identity, sometimes derived from sharing of place of birth of oneself or one’s parents. They are often perceived as sharing a common culture, a set of behaviours and beliefs, determined by upbringing and choice [11]. The people from India, Pakistan, Bangladesh, Sri Lanka, Nepal, Bhutan and Maldives are widely accepted as South Asian by many authors [12]. ‘South Asian’ is a broad ethnic classification with some phenotypic and cultural similarities. However, there is rich diversity within this group with regard to religion, culture and languages. In the linguistic validation process, some similar problems were encountered across the five South Asian languages as well as some individual language specific issues. Ethnicity or ethnic identity may indeed change over time for individuals and mean different things to different people [11]. Because of acculturation, most of the cultural issues would be less of a concern if the questionnaire was administered only to South Asians who are established residents of the UK. However, our goal was to make the translations acceptable for both acculturated and non-acculturated individuals.

Most of the South Asian cultures discuss sexual relationships within the context of a heterosexual marriage. Other sexual relationships such as homosexual or with a sexual partner other than spouse are less accepted. In some countries certain types of sexual relationship and watching adult movies are punishable offences by the established civil/religious law in the country. The participants in the pilot testing pointed out that this could have an effect on the responsiveness and the truthfulness of the response. However the impact of such issues are likely to be minimal for those residing in the UK.

Cross-cultural studies are of considerable importance, and high-quality translations are crucial for this purpose. Linguistic validation as described in this paper is essential in order to develop translated versions. However, in order to allow cross-cultural comparisons, it is necessary to carry out psychometric validation, including factor analysis, to establish whether the factor structures of the specific scales (SIS-1 and 2 and SES) are retained after translation. This requires collection of reasonably sized samples for each of the five languages. We are in the process of doing this with a Sinhalese speaking sample. Other researchers are welcome to use the linguistically validated translations to carry out psychometric validation in the other four Asian languages. This will hopefully result in the potential for doing substantial cross-cultural research based on the Dual Control Model.

It is fair to say that the original items in the SIS/SES questionnaire were not made with the idea of translation into languages from contrasting cultures in mind. It may be difficult to avoid items or concepts that are sensitive in some cultural, religious or legal settings. However, in developing future versions of SIS/SES, or other measures of potential importance for cross-cultural research, these issues should be taken into account. Copies of each of the five translations of the SIS/SES questionnaire are available on request from the first author.

## Conclusion

We applied a rigorous translation method to ensure conceptual equivalence and acceptability of SIS/SES in the five different South Asian languages prior to its utilization in the OSDS. However, to complete the cultural adaptation process, future psychometric validation of the translated versions is required among the different language speakers.

## Competing interests

The authors declare that they have no competing interests.

## Authors’ contributions

LSM, SDJ, STK, SS, JCL and JA made substantial contribution to conception and study design. LSM, PNW and PR were involved in data collection. LSM, PNW, SDJ, STK, SS, JCL and JA were involved in refining the study design, statistical analysis and drafting the manuscript. LSM, PR and PNW critically revised the manuscript. All authors read and approved the final manuscript.
